# Antibacterial activity of *Krameria lappacea* root extract against gram-positive and gram-negative bacteria and its cytotoxicity on lung and breast cancer cell lines

**DOI:** 10.3389/fmicb.2025.1662564

**Published:** 2025-11-11

**Authors:** Rewaida Abdel-Gaber, Mohammed Albeshr, Mohamed A. Dkhil, Nada Almohawis, Kareem A. Abdelmeguid, Denis Delic, Saleh Al Quraishy, Esam M. Al-Shaebi

**Affiliations:** 1 Department of Zoology, College of Science, King Saud University, Riyadh, Saudi Arabia; 2 Department of Zoology and Entomology, Faculty of Science, Helwan University, Cairo, Egypt; 3 Department of Chemistry, College of Science, King Saud University, Riyadh, Saudi Arabia; 4 Botany and Microbiology Department, Faculty of Science, Helwan University, Cairo, Egypt; 5 Translational Medicine and Clinical Pharmacology, Boehringer Ingelheim Pharma GmbH & Co. KG, Biberach, Germany

**Keywords:** *Krameria lappacea*, phytochemical screening, antibacterial activity, gram-bacteria, cytotoxicity

## Abstract

**Introduction:**

The rise of antibiotic-resistant microbes has diminished antibiotic effectiveness, leading to the exploration of alternatives. Krameria lappacea has been used traditionally for various ailments. This study evaluates the in vitro antibacterial and cytotoxic potential of its roots and identifies its active constituents.

**Methods:**

Roots of K. lappacea were acquired from a Riyadh market. They were extracted using methanol and the maceration method, followed by phytochemical screening via Liquid Chromatography-Electrospray Ionization-Mass Spectrometry. Antibacterial properties were assessed using agar well diffusion and broth microdilution methods, while cytotoxicity was tested on human lung A549 and MCF7 breast cancer cell lines via MTT assay.

**Results:**

Phytochemical analysis of the extract identified alkaloids, phenolics (including anthraquinones and chlorogenic acids), and flavonoids (such as dihydrokaempferol, epicatechin, and fisetin). The most susceptible bacteria were Staphylococcus aureus and Listeria monocytogenes, both Gram-positive, while Gram-negative Escherichia coli and Salmonella enterica were resistant. Extract effectively suppressed bacterial growth, particularly against L. monocytogenes and S. aureus, showing moderate activity against E. coli and S. enterica, with MBC values higher than MICs. Cytotoxicity testing yielded IC50 values of 142.27 ± 1.02 μg/ml and 64.81 ± 0.26 μg/ml, respectively, compared to doxorubicin.

**Conclusion:**

Our findings suggest that K. lappacea possesses notable antibacterial activity against Gram-positive bacteria and demonstrates cytotoxic effects against human cancer cell lines, indicating its potential as a natural source of bioactive compounds for antimicrobial and anticancer applications.

## 1 Introduction

Herbal medicine has been a cornerstone in the treatment of various ailments for centuries (Maciel et al., 2002). The World Health Organization (WHO) defines herbal medicine as a practice encompassing herbs, herbal materials, herbal preparations, and finished herbal products that contain active ingredients derived from plants or other plant materials, or their combinations (Robinson and Zhang, 2011; Chaachouay and Zidane, 2024). The therapeutic value of these plants is attributed to secondary metabolites, which are organic compounds not directly involved in the plant’s growth, development, or reproduction but play crucial roles in the plant’s defense against pathogens and herbivores (Andrade et al., 2007; Ansari et al., 2023). These metabolites include alkaloids, flavonoids, terpenoids, and phenolics, which have been shown to possess anti-inflammatory, antimicrobial, anticancer, and antioxidant properties (Chaachouay and Zidane, 2024). In recent years, there has been a growing trend toward seeking herbal alternatives due to their accessibility, safety, and affordability. Studies have highlighted the neuroprotective and metabolic regulatory functions of plant-based secondary metabolites, underscoring their potential for therapeutic use (El-Shouny et al., 2018; Ilaghi et al., 2021; Abdel-Gaber et al., 2024; Khanam et al., 2025).

*Krameria lappacea*, commonly known as rhatany, is a slow-growing shrub belonging to the Krameriaceae family. The roots are rich in diverse bioactive compounds, including phenolics, flavonoids, lignan derivatives, tannins, benzofuran derivatives, and oligomeric proanthocyanidins, which contribute to its pharmacological benefits (Aldakheel et al., 2021; Safta et al., 2023; Abdel-Gaber et al., 2024). Earlier phytochemical investigations have reported the presence of flavonoids and oligomeric proanthocyanidins as the dominant constituents (Scholz and Rimpler, 1989; Genovese et al., 2021; Zabka, 2022), which provide a strong basis for subsequent pharmacological exploration. Traditionally, *K. lappacea* has been used to treat gastrointestinal disorders (Simpson, 1989) and is valued for its anti-inflammatory and anti-hemorrhagic properties, making it useful in conditions such as stomach ailments, oropharyngeal inflammation, and excessive blood loss (Simpson, 1991; Carini et al., 2002; Safta et al., 2023; Abdel-Gaber et al., 2024). More recently, a wide range of pharmacological activities have been reported, including photoprotective (Carini et al., 2002), antidiabetic (Heiss et al., 2012), vasoprotective (Ladurner et al., 2012), antimicrobial (Bombardelli et al., 2002; Ortiz et al., 2019; Genovese et al., 2021; Zabka, 2022), antibacterial (Scholz and Rimpler, 1989; Ortiz et al., 2019; Safta et al., 2023), anticancer (Al-Oqail, 2021), anticoccidial (Alamari et al., 2024), and insecticidal (Al-Fuhaid, 2018) activities.

This study aims to comprehensively assess the effectiveness of the methanolic extract derived from the roots of *K. lappacea*. The research will focus on three primary objectives: (i) Identification and characterization of active constituents present in the methanolic extract. (ii) Evaluation of the antibacterial activity of the extract against both Gram-positive and Gram-negative bacterial strains. (iii) Assessment of the cytotoxic potential of the *K. lappacea* extract against human lung cancer cell lines, specifically A549, and breast cancer cell lines, namely MCF7. Through these comprehensive evaluations, the study aims to contribute valuable knowledge regarding the pharmacological benefits of *K. lappacea* and its potential use in medicinal applications.

## 2 Materials and methods

### 2.1 Plant collection and extract preparation

The roots of *K. lappacea* were obtained from a local market in Riyadh, Saudi Arabia. A qualified taxonomist, Prof. Mohamed A. El-Sheikh, authenticated the plant specimen, which was assigned the voucher number KSU-22958, at the herbarium of the Botany Department, College of Science, King Saud University (Saudi Arabia). Authentication was based on a detailed examination of the plant’s morphological traits (e.g., root structure, color, and texture) in comparison with standard taxonomic references and databases. While the present identification relied on morphological features, we acknowledge that anatomical analysis of root tissues (e.g., transverse sections, xylem and phloem structure, and histological features) would provide an additional confirmatory layer, and we intend to incorporate such analysis in future work.

The extract of the *K. lappacea* roots was carried out using maceration following the method described by Manikandan et al. (2008). Briefly, the roots were broken into smaller pieces and then coarsely powdered using a Hummer grinder (ED-CG1400). The dried and powdered roots (100 g) were soaked in 1,000 mL of 70% methanol at room temperature for 24 h with occasional stirring. The mixture was then filtered through Whatman No. 1 filter paper, and the residue was re-macerated twice under the same conditions to ensure maximum extraction of phytochemicals. The combined filtrates were concentrated under reduced pressure using a rotary vacuum evaporator, specifically a Buchi model from Switzerland, at 55 °C and subsequently dried to yield the crude extract. The extract was stored at −20 °C until further analysis.

### 2.2 Liquid chromatography-electrospray ionization-mass spectrometry (LC-ESI-MS)

LC-ESI-MS was employed to identify the phytochemicals in the extract, in triplicate, to provide a comprehensive profile relevant to its pharmacological properties. The analysis was performed using an XEVO TQD triple quadrupole mass spectrometer equipped with an electrospray ionization (ESI) source (Waters Corporation, Milford, MA, USA). Data were acquired in both positive and negative ionization modes. Chromatographic separation was carried out on an ACQUITY UPLC BEH C18 column (2.1 × 50 mm, 1.7 μm particle size) maintained at 40 °C, with a flow rate of 0.2 mL/min. The mobile phase consisted of (A) water containing 0.1% formic acid and (B) acetonitrile containing 0.1% formic acid, using a linear gradient program optimized for phytochemical separation. For mass spectrometric detection, the capillary voltage was set at 3.0 kV, cone voltage at 30 V, desolvation temperature at 350 °C, source temperature at 120 °C, and desolvation gas (nitrogen) flow at 800 L/h. Mass spectra were collected over an *m*/*z* range of 100–1,200 with a scan time of 0.2 s. Compound identification was achieved by comparing experimental mass spectra and fragmentation patterns with reference spectra from the Waters UNIFI Scientific Information System and cross-verified using the MassBank and METLIN databases. A matching score threshold of ≥85% similarity was applied for tentative identifications, while compounds with ≥95% match were considered high-confidence identifications. Retention times and fragmentation consistency were additionally considered to confirm compound identity and minimize false positives. This combined approach—utilizing spectral matching, retention time verification, and fragmentation pattern analysis—ensured a robust and reproducible identification of the phytochemicals present in the extract.

### 2.3 Microorganisms

The test organisms were sourced from the American Type Culture Collection (ATCC, Manassas, USA) and included two Gram-positive bacteria: *Listeria monocytogenes* (ATCC 7644) and *Staphylococcus aureus* (ATCC 25923), as well as two Gram-negative bacteria: *Escherichia coli* (ATCC 8739) and *Salmonella enterica* (ATCC 14028).

### 2.4 Evaluation of the antibacterial potential of the methanolic extract

The experiment was conducted using the agar well-diffusion method, following the guidelines set by the Clinical and Laboratory Standards Institute (CLSI, 2012, 2019), Gholizadeh et al. (2013), and Balouiri et al. (2016). In summary, 100 μL of each reference strain (1 × 10^5^ colony-forming unit (CFU)/mL) was separately spread over the Mueller-Hinton Agar (MHA) medium. After the medium had fully solidified, we utilized a sterile cork borer to carefully create wells with a precise diameter of 0.6 cm. These wells were designed to accommodate 50 μL of different concentrations of the methanolic extract, which varied from 0 to 133 mg/mL. The plates were first placed in the refrigerator for 30 min to enhance the diffusion of the sample extracts into the agar (Gonelimali et al., 2018; Eloff, 2019). After this period, the plates were incubated at 37 °C for 24 h. The antibacterial potential of each sample was assessed by measuring the diameter of the zones of inhibition (ZOI) in millimeters (mm), using three replicates. This activity was compared to that of several conventional antibiotics with varying modes of action: amikacin (AK, 30 μg/mL) as a protein synthesis inhibitor; amoxicillin (AX, 25 μg/mL) and ampicillin/sulbactam (SAM, 20 μg/mL) as cell wall synthesis inhibitors; norfloxacin (NOR, 10 μg/mL) and ofloxacin (OFX, 5 μg/mL) as DNA replication inhibitors. Sterile dimethyl sulfoxide (DMSO) of 50 μL was utilized as a negative control.

### 2.5 Determination of minimum inhibitory concentrations (MICs)

The broth microdilution assay was used to determine the minimum inhibitory concentrations (MICs) of each tested sample, following the protocols outlined by EUCAST (2003), Gholizadeh et al. (2013), and CLSI (2012, 2019). A stock solution of the extract was prepared by carefully dissolving 133 mg of the extract in 1 mL of 1% dimethyl sulfoxide (DMSO). This process involved gradually adding the extract to the DMSO while stirring gently to ensure complete dissolution. The resulting solution was then thoroughly mixed to achieve a homogeneous stock solution suitable for further experimental use. Following this, 100 μL of Mueller-Hinton Broth (MHB) was added to each well from wells 2–12. Then, 150 μL of the stock solution was added to the first column of a microtiter plate. A two-fold dilution was performed by transferring 100 μL from the first well to the next, continuing this process until the 11th well. In the final step, 100 μL of each microbial culture (1 × 10^5^ CFU/mL) was added to each well, except the last one, which served as the blank control. All microtiter plates were incubated at 37 °C for 24 h for the inoculated bacterial strains. Absorbances were measured at a wavelength of 620 nm using an automated microplate reader (ChroMate 4,300, United States), and the results were graphically represented using Microsoft Excel version 2019. Statistical significance was determined with a *p*-value of < 0.05 compared to the control group. Chloramphenicol (1 mg/mL) and ciprofloxacin (1 μg/mL) were used as positive controls for antibacterial activity.

### 2.6 Assessment of the minimum bactericidal concentration (MBC)

The minimum bactericidal concentration (MBC) for the extract was determined using the dilution in broth method, following the guidelines set by CLSI (2019). Aliquots of 50 μL were taken from all wells that showed no visible growth after 24 h of incubation at 37 °C and were spread onto Mueller-Hinton agar (MHA) plates. These plates were then incubated for an additional 24 h at 37 °C. The limit of detection (LOD) for this technique is 10 CFU/mL; therefore, the absence of any growth on the MHA plates indicated that the concentration was below this threshold. This implies that the initial concentration of 10^5^ CFU/mL had been significantly reduced to a level below 10 CFU/mL. Subsequently, the MBC was assessed as the minimum concentration of the extract capable of killing more than 99.99% of the bacteria present. Three replicates of each trial were conducted.

### 2.7 Cytotoxicity assay

The cytotoxicity of the extract was evaluated, in triplicate, using the MTT assay, specifically with 3-(4,5-dimethylthiazol-2-yl)-2,5-diphenyltetrazolium bromide, according to Mosmann (1983). The assay was applied to human lung (A549) and breast (MCF7) cancer cell lines obtained from ATCC (USA). The cells were cultured in Dulbecco’s Modified Eagle Medium (DMEM) (Gibco, USA) supplemented with 1% penicillin/streptomycin and 10% fetal bovine serum (FBS) (Gibco, USA). They were incubated at 37 °C in a humidified atmosphere containing 5% CO_2_ for 24 h in a CO_2_ incubator (LCO-065AI, Daihan LabTech Co., LTD). In summary, cells were plated in a 96-well culture plate at a density of 2 × 10^5^ cells/ml and allowed to proliferate for 24 h. The cells were treated with various extract concentrations (500, 250, 125, 62.5, 31.25, and 15.625 μg/mL) and doxorubicin. After treatment, 20 μL of MTT solution (5 μg/mL in PBS) was added to each well, and the plate was incubated for 4 h at 37 °C. The yellow tetrazolium salt was reduced to purple formazan crystals, which were then dissolved in 100 μL of DMSO. The absorbance of the resulting colored solution was measured spectrophotometrically at 570 nm on a microplate reader (BioTek, USA). Cell viability (%) was calculated following the method described by Shirley and Lillehoj (2012). The doxorubicin-treated cells served as a positive control. IC_50_ values (the concentration of extract that causes 50% inhibition) were calculated from the dose–response curve of cell viability percentages using OriginPro software.

## 3 Results and discussion

The phytochemical profile obtained in this study revealed several compounds, including flavonoids and oligomeric proanthocyanidins, which are consistent with previous reports on *K. lappacea* (Scholz and Rimpler, 1989; Genovese et al., 2021; Zabka, 2022). However, some detected compounds, such as sanguinarine, an alkaloid, which have not been previously reported in this species and were identified tentatively based on MS/MS fragmentation patterns. Its identification in the present study was based on MS/MS fragmentation and database matching; however, confirmation requires further characterization using authentic standards, multiple reaction monitoring (MRM), and complementary structural analyses (e.g., NMR). Therefore, sanguinarine is reported here as a preliminary finding that warrants validation in future investigations. Previous studies by Yan et al. (2021) and Ardhany et al. (2022) have shown that sanguinarine exhibits potent antibacterial activity through multiple mechanisms, including disruption of bacterial membranes, DNA intercalation, induction of reactive oxygen species (ROS), and inhibition of essential bacterial enzymes, ultimately leading to bacterial cell death. Recent research by Gu et al. (2023) further corroborates these findings, demonstrating that sanguinarine chloride hydrate (SGCH) effectively compromises the cell wall and membrane integrity of *Staphylococcus aureus*, inducing oxidative stress and bacterial lysis.

In addition to sanguinarine, six flavonoids were detected, each associated with specific biological activities. Eriodictyol possesses antioxidant, anti-inflammatory, neuroprotective, and anticancer properties (Deng et al., 2020). Dihydrokaempferol demonstrates *in vitro* antimicrobial activity against diverse microorganisms (Elmongy et al., 2022). Isobavachin has broad-spectrum pharmacological effects, including antimicrobial and cytotoxic actions (Chung et al., 2024). Epicatechin exerts antioxidant, antimicrobial, anti-inflammatory, and anticancer effects by scavenging ROS, disrupting bacterial membranes, and inducing apoptosis in cancer cells (Prakash et al., 2019). Fisetin has been shown to inhibit cancer cell proliferation, induce apoptosis, suppress angiogenesis, and protect against oxidative stress (Zhou et al., 2023). Naringenin exhibits significant antioxidant, antimicrobial, and anti-inflammatory activity, contributing to both antibacterial and anticancer effects (Moon et al., 2011; Wang et al., 2016). Recent reviews by Zhang et al. (2025) and Liu et al. (2025) have highlighted the antibacterial potential of flavonoids, emphasizing their mechanisms, including membrane disruption, inhibition of nucleic acid and protein synthesis, and suppression of efflux pumps.

Two phenolic compounds—chlorogenic acid and anthraquinones—were also identified. Chlorogenic acid is known for antibacterial, antioxidant, anti-inflammatory, and anticancer activities, functioning through mechanisms such as ROS scavenging, disruption of bacterial cell walls, and induction of apoptosis in tumor cells (Miao and Xiang, 2020). Anthraquinones are recognized for antimicrobial and anti-inflammatory properties and can compromise bacterial membrane integrity (Malik and Müller, 2016). These findings are consistent with those of Safta et al. (2023), who identified similar compounds in *K. lappacea* root extracts and attributed their bioactivity to these constituents. Each compound exhibited distinct peak areas and retention times, which were carefully documented in Table 1.

**Table 1:** Phytochemical compounds were identified from the *K. lappacea* roots extract using LC-ESI-MS in both positive and negative ionization modes.

Compound	RT	MW (g/mol)	Ionization mode	Total area %	Chemical formula	Chemical structure	Compound family
Sanguinarine	0.89	332.3	[M + H]+	1.41	C_20_H_14_NO_4_	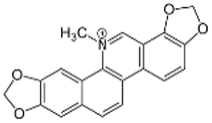	Alkaloid
Eriodictyol	5.64	288.25	[M – H]−	2.08	C_15_H_12_O_6_	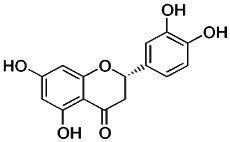	Flavonoid
Dihydrokaempferol (Aromadendrin)	7.30	288.25	[M – H]−	2.58	C_15_H_12_O_6_	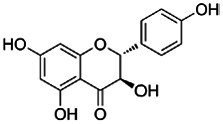	Flavonoid
Isobavachin	9.14	324.4	[M – H]−	2.00	C_20_H_20_O_4_	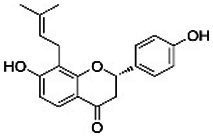	Flavonoid
Epicatechin	9.71	290.27	[M – H]−	7.30	C_15_H_14_O_6_	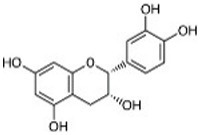	Flavonoid
Fisetin	11.10	286.24	[M + H]+	1.90	C_15_H_10_O_6_	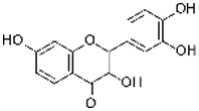	Flavonoid
Chlorogenic acid	12.30	354.31	[M + H]+	1.24	C_16_H_18_O_9_	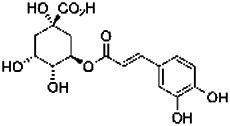	Phenolics
Naringenin	16.60	272.25	[M + H]+	1.38	C_15_H_12_O_5_	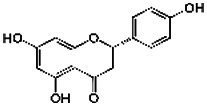	Flavonoid
Anthraquinone	27.30	208.21	[M – H]−	0.40	C_14_H_8_O_2_	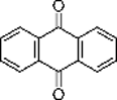	Phenolics

Identification was based on comparison of experimental MS/MS fragmentation patterns and retention times with reference spectra in the Waters UNIFI Scientific Information System, MassBank, and METLIN databases. A similarity score threshold of ≥85% was applied for tentative identification, and compounds with ≥95% match were considered high-confidence identifications.

The antibacterial activity of *K. lappacea* extract is likely mediated through several complementary pathways. Flavonoids and related phytochemicals can disrupt bacterial cell walls and membranes, causing leakage of intracellular contents and compromising structural integrity (Genovese et al., 2021). Some compounds intercalate with bacterial DNA, hindering replication and transcription (Abdel-Gaber et al., 2024). Others bind to bacterial enzymes and proteins, impairing energy metabolism, protein synthesis, and cell wall biosynthesis. Certain constituents may also interfere with quorum-sensing pathways, reducing biofilm formation and inducing ROS-mediated oxidative stress, collectively leading to bacterial lysis (Genovese et al., 2021; Abdel-Gaber et al., 2024). Collectively, these mechanisms act synergistically to inhibit bacterial growth and ultimately cause bacteriolysis.

**Figure 1:**
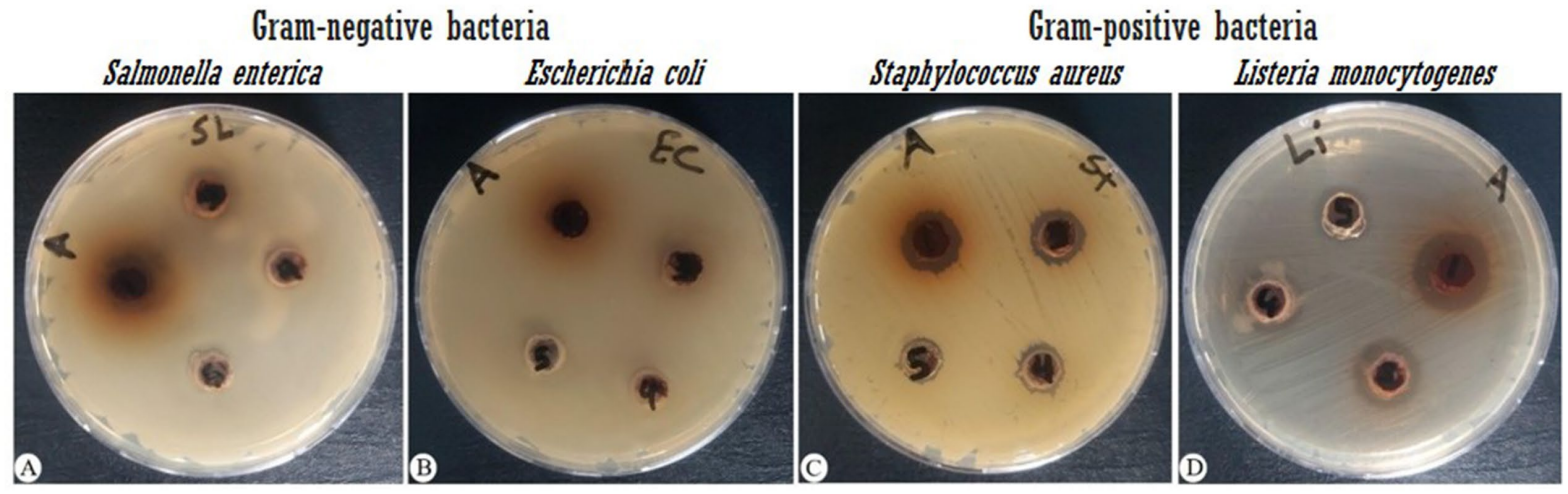
Antibacterial activity of *K. lappacea* methanolic extract against four bacterial strains, measured as the zone of inhibition (mm). Extract concentrations tested were 133, 66.7, 33.3, 16.7, and 8.3 mg/mL. **(A)** *Salmonella enterica* (ATCC 14028), **(B)** *Escherichia coli* (ATCC 8739), **(C)** *Staphylococcus aureus* (ATCC 25923), and **(D)** *Listeria monocytogenes* (ATCC 7644).

In agar diffusion assays, the extract produced a ZOI of 14 mm against *S. aureus* and 19 mm against *L. monocytogenes* (Figure 1; Table 2). This aligns partially with Babiker et al. (2020), who reported a 19 mm ZOI for *Krameria triandra* against *S. aureus*, highlighting species-specific differences in bacterial sensitivity potentially linked to variations in the phytochemical profile and structure–activity relationships of the extracts. The stronger effect against *L. monocytogenes* may reflect differences in cell wall composition and permeability among Gram-positive bacteria (Abdel-Gaber et al., 2024). In contrast, Gram-negative bacteria (*E. coli* and *S. enterica*) were resistant, likely due to the protective outer membrane and lipopolysaccharide layer, which restricts entry of hydrophobic phytochemicals (Inouye et al., 2001; Horne et al., 2020; Salem et al., 2023). This resistance was consistent with Nayf and Salman (2021), who observed larger inhibition zones with standard antibiotics (such as Amikacin, Amoxicillin, Ampicillin/sulbactam, Norfloxacin, and Ofloxacin) compared to plant extracts. Overcoming Gram-negative resistance may require combining phytochemicals with agents that disrupt the outer membrane or inhibit efflux pumps, thereby improving intracellular accumulation of active compounds. Several studies have reported such effects: Wesseling and Martin (2022) highlighted peptide-based permeabilizers that sensitize bacteria to antimicrobials, Duffey et al. (2024) catalogued numerous efflux pump inhibitors in *E. coli* and *Pseudomonas*, and Al-Sallami et al. (2023) demonstrated that plant extracts like rosemary, clove, and cumin can potentiate antibiotic activity via efflux pump inhibition. Similarly, Minnelli et al. (2024) showed that amphiphilic *α*-hydrazido acids permeabilize bacterial membranes and act synergistically with antibiotics. Building on these precedents, combining *K. lappacea* extract with safe permeabilizers or efflux pump inhibitors could broaden its spectrum against Gram-negative pathogens, a hypothesis that warrants validation through synergistic assays, intracellular accumulation studies, and toxicity assessments.

**Table 2:** Antibacterial susceptibility test results of the extract.

Sample	Extract				AK	AX	SAM	NOR	OFX
	(mg mL^−1^)				(μg/mL)				
Concentration μg mL^−1^	133	33.3	16.7	8.3	30	25	20	10	5
Gram-negative bacteria									
*S. enterica* ATCC 14028	N/D	N/D	N/D	N/D	21	21	10	35	30
*E. coli* ATCC 8739	N/D	N/D	N/D	N/D	22	N/D	19	30	29
Gram-positive bacteria									
*S. aureus* ATCC 25923	14	13	12	9	22	21	24	25	27
*L. monocytogenes* ATCC 7644	19	16	14	12	7	7	7	8	7

N/D not detected, Results represent the mean diameter of the clear zone of inhibition in millimeters (mm) for three replicates diameter (mm) of each trial; Amikacin (AK 30), protein synthesis inhibitor; Amoxicillin (AX 25), and Ampicillin/sulbactam (SAM 20), cell wall synthesis inhibitors, Norfloxacin (NOR 10) and Ofloxacin (OFX 5), DNA replication inhibitor; Diluted DMSO was used as a negative control.

**Figure 2:**
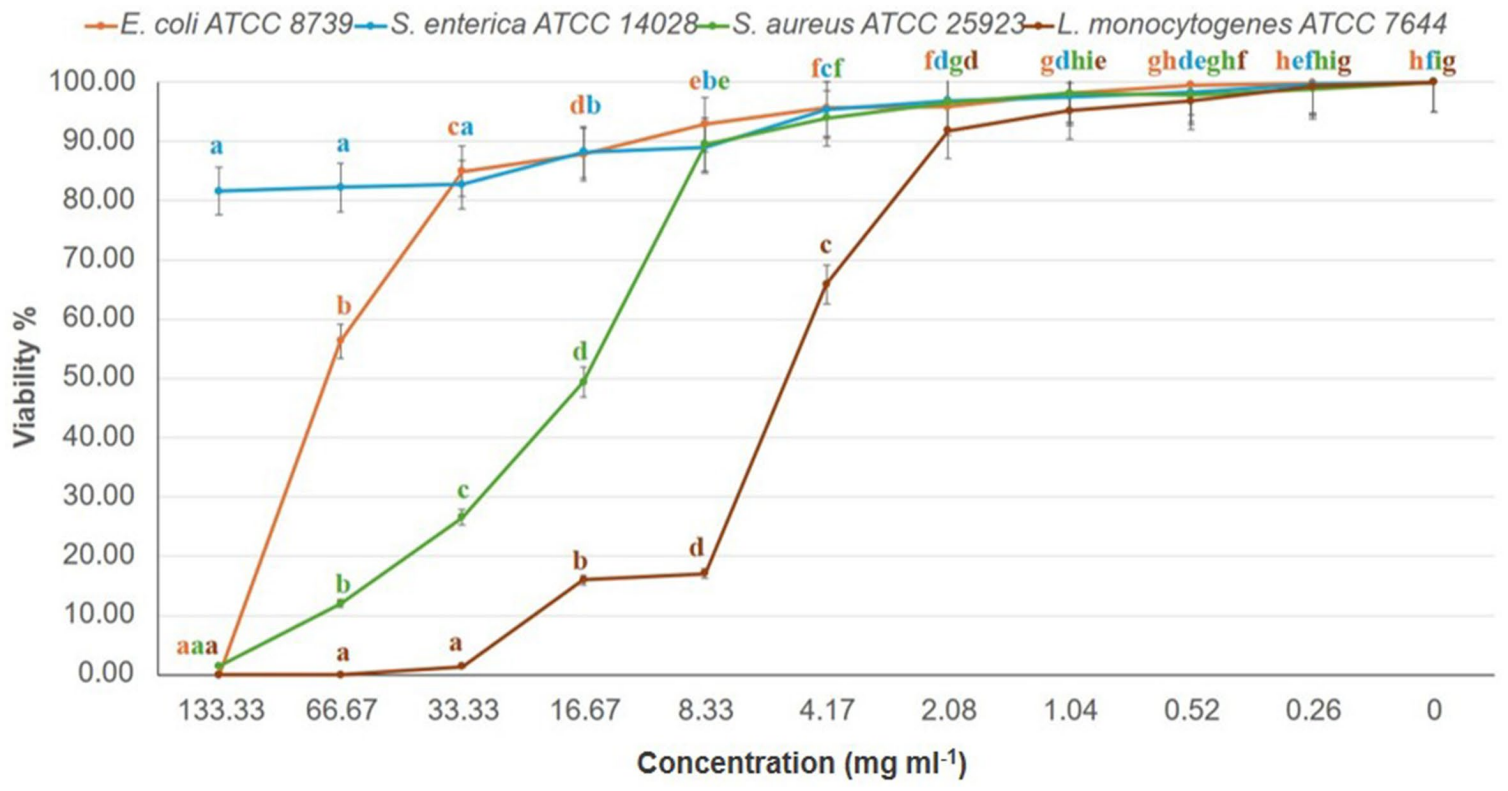
Dose–response effect of *K. lappacea* methanolic extract on the viability (%) of four bacterial reference strains—*Salmonella enterica* (ATCC 14028), *Escherichia coli* (ATCC 8739), *Staphylococcus aureus* (ATCC 25923), and *Listeria monocytogenes* (ATCC 7644)—assessed using the broth dilution assay. Extract concentrations tested were 133, 66.7, 33.3, 16.7, and 8.3 mg/mL. Error bars represent standard deviations from three independent experiments (n = 3). Means with different letters are significantly different (*p* < 0.0001) according to one-way ANOVA followed by Duncan’s *post hoc* test.

MIC and MBC analyses revealed effective inhibition of Gram-positive strains, particularly L. monocytogenes (MIC = 67 mg/mL) and S. aureus (MIC = 133 mg/mL), with MBCs ranging from 34 to 67 mg/mL (Figure 2; Table 3). These results are supported by Bouarab-Chibane et al. (2019) and Hochma et al. (2021), highlighting the susceptibility of Gram-positive bacteria to phenolic compounds due to their simpler cell wall structure. In line with our results, Al-Oqail (2021) investigated the antibacterial and cytotoxic activities of *K. lappacea* root extracts. Their findings revealed that the ethanolic extract exhibited inhibitory effects against both Gram-positive and Gram-negative bacteria, with MIC values ranging from 250 to 1,000 μg/mL. Mendes et al. (2022) highlighted that phenolic compounds, such as anthraquinones and chlorogenic acids, act by disrupting peptidoglycan cross-links, leading to leakage of proteins and phospholipids, thereby compromising membrane integrity and causing bacterial death. In contrast, certain flavonoids, such as dihydrokaempferol-3-O-α-L-rhamnoside, have been reported to overcome Gram-negative barriers depending on chemical structure (Elmongy et al., 2022). Alkaloids, including sanguinarine, further enhance antibacterial efficacy by compromising membranes, inhibiting DNA and protein synthesis, and inducing ROS-mediated stress (Yan et al., 2021; Ardhany et al., 2022). Taken together, the present results reinforce the antimicrobial potential of *K. lappacea*, while comparative studies highlight the importance of both bacterial cell wall composition and specific phytochemical structures in determining susceptibility.

**Table 3:** Minimum inhibitory concentrations (MICs) and minimum bactericidal concentrations (MBCs) of the extract.

Bacterial reference strains	Concentrations mg mL^−1^			
	MIC	MBC	C	CIP
Gram-negative bacteria				
*S. enterica* ATCC 14028	>133	>133	0.25	0.25
*E. coli* ATCC 8739	>133	>133	0.5	0.25
Gram-positive bacteria				
*S. aureus* ATCC 25923	133	67	0.25	0.5
*L. monocytogenes* ATCC 7644	67	34	0.5	0.25

*P*-value * < 0.000001 s. MIC antibacterial positive controls: Chloramphenicol (C) and ciprofloxacin (CIP).

The cytotoxic activity of the *K. lappacea* extract was evaluated against A549 (lung carcinoma) and MCF7 (breast carcinoma) cell lines using the MTT assay (Figure S1; Table 4), showing dose-dependent inhibition of cell viability. The extract demonstrated higher cytotoxicity against MCF7 (IC₅₀ = 64.81 ± 0.26 μg/mL) than A549 (IC₅₀ = 142.27 ± 1.02 μg/mL), whereas doxorubicin—a widely used chemotherapeutic—exhibited substantially greater potency at much lower concentrations. This highlights the relatively moderate activity of the extract compared to established anticancer agents. In line with our results, Al-Oqail (2021) reported that the extract demonstrated cytotoxicity against MCF-7 breast cancer cells, with IC₅₀ values between 8.883 and 3.17 μM. These results align with our study’s observations and provide a broader context for understanding the therapeutic potential of *K. lappacea* extracts. The cytotoxic effects of the extract are attributed to flavonoids (epicatechin, fisetin) and phenolics (chlorogenic acid), which exert their activity through multiple mechanistic pathways: induction of apoptosis via caspase activation, mitochondrial dysfunction with loss of membrane potential and cytochrome c release, generation of reactive oxygen species (ROS) causing oxidative stress, interference with cell cycle progression, and inhibition of oncogenic signaling pathways such as PI3K/Akt and MAPK (Govarthanan et al., 2016; Prakash et al., 2019; Miao and Xiang, 2020; Zhou et al., 2023). These mechanisms collectively reduce proliferation and promote selective cancer cell death. Importantly, while the extract demonstrates only moderate potency compared with doxorubicin and reference antibiotics (e.g., ampicillin and vancomycin), such findings underscore the potential of exploring synergistic strategies. Previous studies have shown that combining phytochemicals with conventional chemotherapeutics or antibiotics can enhance therapeutic efficacy, reduce required drug dosages, and overcome resistance mechanisms (Wesseling and Martin, 2022). Future investigations should therefore assess whether *K. lappacea* root extract, or its bioactive constituents, can act synergistically with standard anticancer agents like doxorubicin or with frontline antibiotics. Such studies would not only clarify the clinical relevance of our findings but also open new avenues for the rational development of combination therapies.

**Table 4:** IC_50_ of the studied extract.

Sample	Cell lines and IC_50_ (μg/mL)	
	A549	MCF7
Extract	142.27 ± 1.02	64.81 ± 0.26
Doxorubicin	1.55 ± 0.05	0.95 ± 0.01

Overall, LC-ESI-MS analysis links specific alkaloids and flavonoids to their reported antibacterial and cytotoxic mechanisms, providing a comprehensive rationale for the extract’s multifactorial biological activity. These findings reinforce *K. lappacea*’s potential as a source of bioactive compounds for antimicrobial and anticancer applications, while highlighting that efficacy is influenced by phytochemical composition, target cell type, and the inherent sensitivity of bacterial or cancer cells relative to established reference agents like doxorubicin and standard antibiotics.

## 4 Conclusion

Preliminary *in vitro* findings indicate that *K. lappacea* may possess bacteriostatic activity against Gram-positive bacteria and exhibit cytotoxic effects on human lung and breast (MCF7) cancer cell lines. However, subsequent studies will focus on identifying precise molecular targets (e.g., bacterial cell wall biosynthesis, efflux pump inhibition, apoptosis, or cell cycle arrest in cancer cells) and delineating signaling pathways through proteomic, transcriptomic, and molecular docking approaches.

## Impact statement

*Krameria lappacea* demonstrates promising bacteriostatic activity against Gram-positive bacteria, suggesting potential as a complementary or alternative strategy to conventional antibiotics. It also exhibits cytotoxic effects against human lung (A549) and breast (MCF7) cancer cells, supporting its relevance as a source of bioactive compounds with anticancer potential. While further studies are required to elucidate its mechanisms of action and validate therapeutic applications, the consistency between its traditional use and the present findings highlights its potential role in developing novel antimicrobial and anticancer agents.

## Data Availability

The raw data supporting the conclusions of this article will be made available by the authors, without undue reservation.
