# Supplementary material for: Antibacterial activity of Krameria lappacea root extract against gram-positive and gram-negative bacteria and its cytotoxicity on lung and breast cancer cell lines
Source: Front Microbiol. 2025 Nov 11;16:1662564. doi: 10.3389/fmicb.2025.1662564 (PMC12645635; doi:10.3389/fmicb.2025.1662564)
Supplement: Supplementary file 1 [file Data_Sheet_1.docx]

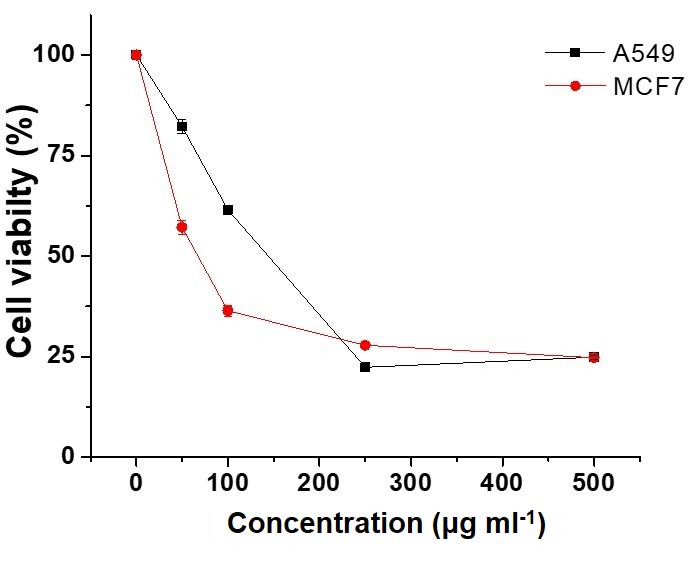


**Figure S1.** MTT assay for testing extract cytotoxicity at various concentrations (μg ml^-1^) against lung (A549) and breast (MCF7) cancer cell lines after 48 hr of incubation.
